# Laparoscopic spleen-preserving distal pancreatectomy for epidermoid cyst in an intrapancreatic accessory spleen: A case report

**DOI:** 10.1097/MD.0000000000026379

**Published:** 2021-07-02

**Authors:** Xiang Zheng, Bo Zhou, Jing-Qing Sun, Ming Jin, Sheng Yan

**Affiliations:** a Department of General Surgery, Second Affiliated Hospital, School of Medicine, Zhejiang University, Hangzhou 310009; b Affiliated Hospital of Stomatology, School of Stomatology, Zhejiang University School of Medicine, and Key Laboratory of Oral Biomedical Research of Zhejiang Province, Hangzhou 310006, Zhejiang Province, China.

**Keywords:** epidermoid cyst, intrapancreatic accessory spleen, laparoscopic distal pancreatectomy

## Abstract

**Rationale:**

Pancreatic tail cystic lesions are increasingly encountered in clinical practice, however, it is difficult to make a correct diagnosis preoperatively because there are many types of pancreatic neoplastic and non-neoplastic cysts. Epidermoid cyst in an intrapancreatic accessory spleen (ECIPAS) is a rare non-neoplastic cyst locating in the pancreatic tail, and it is commonly misdiagnosed as another cystic neoplasm.

**Patient concerns:**

A 51-year-old man was admitted for investigation of abdominal pain. The physical examination and laboratory tests found no abnormalities, except for an elevation of carbohydrate antigen (CA)19-9. Imaging revealed a cystic lesion within the pancreatic tail, and the solid component surrounding the cyst was enhanced similarly to those of the splenic tissue.

**Diagnosis:**

ECIPAS was diagnosed based on the pathology after surgery. The mass was composed of a cyst and brown solid spleen-like tissue. The microscopic analysis demonstrated that the solid component was accessory splenic tissue, and the cyst wall was lined with a thin stratified squamous epithelium.

**Interventions:**

Laparoscopic spleen-preserving distal pancreatectomy was performed.

**Outcomes:**

The patient was discharged on day 5 postoperatively after an uneventful recovery. CA19-9 returned to normal after surgery. During a 2-years follow-up, there was no evidence of tumor recurrence.

**Lessons:**

Although rare ECIPAS should be considered in the differential diagnosis of pancreatic tail cystic lesions, and the typical imaging features might facilitate the preoperative diagnosis. Laparoscopic distal pancreatectomy is a safe and effective approach for treating ECIPAS.

## 1 Introduction

Although pancreatic tail cystic lesions are being increasingly encountered in clinical practice, it is difficult to make an exact diagnosis preoperatively.^[1–3]^ Epidermoid cyst in an intrapancreatic accessory spleen (ECIPAS) is an exceedingly rare entity, and all the ECIPAS cases reported so far have been found in the pancreatic tail.^[4,5]^ ECIPAS should therefore be considered in the differential diagnosis of pancreatic tail cystic lesions. It is difficult to diagnose ECIPAS preoperatively using conventional imaging, thus it is commonly misdiagnosed as another cystic neoplasm, such as a mucinous cystic neoplasm (MCN), solid pseudopapillary tumor (SPT), intraductal papillary mucinous neoplasm (IPMN), or cystadenocarcinoma.^[6–9]^ ECIPAS was thought to be benign until recently when it was found to develop into a malignant tumor during 6-years follow-up,^[10]^ which highlights its malignant potential. Thus, it would be necessary to make a definitive diagnosis of this disease as well as to differentiate it from other potentially malignant pancreatic tail cystic neoplasms. We describe a case that was speculated to be ECIPAS, treated by laparoscopic spleen-preserving distal pancreatectomy.

## 2 Case presentation

**Figure 1:**
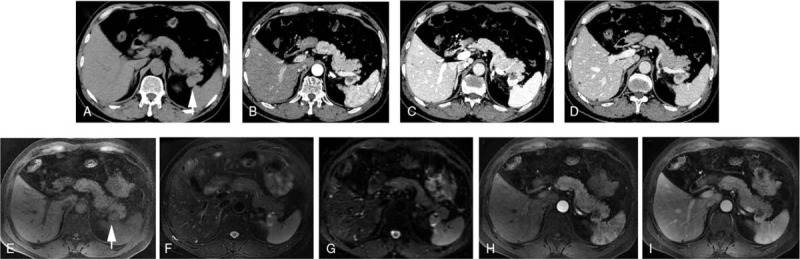
A well-defined cystic lesion (arrow) is revealed in the pancreatic tail. The Contrast-enhanced CT (A–D) and contrast-enhanced magnetic resonance imaging (E–I) demonstrate the enhanced pattern in all phases. (A) Precontrast CT; (B) arterial phase; (C) portal phase; (D) delayed phase; (E) T1-weighted imaging; (F) T2-weighted imaging; (G) diffusion-weighted imaging; (H) arterial phase; and (I) portal phase. CT = computed tomography

**Figure 2:**
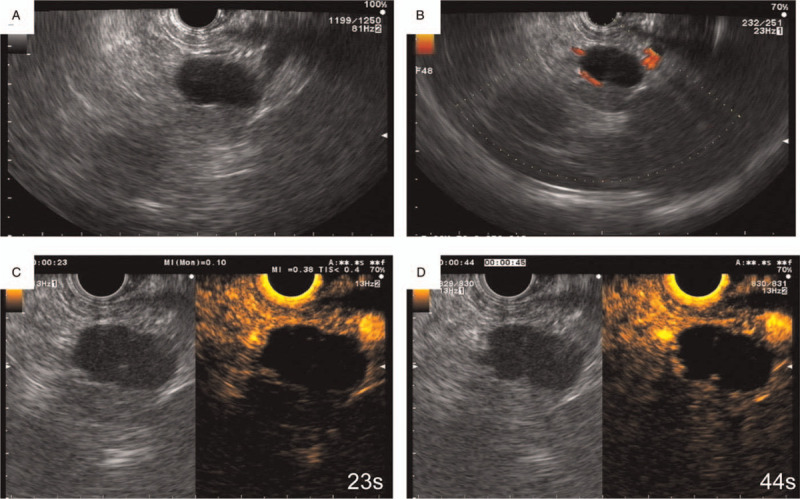
EUS images showing a pancreatic mass located in the tail. (A) B-mode and (B) color Doppler. Contrast-enhanced EUS demonstrating the different time (C: 23 s and D: 44 s) of blood perfusion. EUS = endoscopic ultrasound

A 51-year-old man was admitted to our hospital with a history of abdominal pain for 6 months. The patient had no symptoms of fever, nausea, vomiting, or weight loss. The patient's symptoms started 6 months ago, with recurrent episodes of abdominal pain that could relieve spontaneously. No history of trauma or pancreatitis was recorded. The physical and laboratory examinations were normal, except for elevation of carbohydrate antigen (CA)19-9 to 55 U/mL (normal range 0–37 U/mL). Contrast-enhanced computed tomography (CT) found a cystic lesion measuring 2.6 cm within the tail of the pancreas, and the thick solid wall surrounding the cyst was enhanced similarly to those in splenic tissue (Fig. 1A–1D). Upon magnetic resonance imaging (MRI), the cyst was hyperintense in T1-weighted imaging and heterogeneous in T2-weighted imaging (Fig. 1E and 1F); therefore, the cystic component was considered to be mucinous or bloody liquid. The solid component showed high signal intensity in diffusion-weighted imaging and high intensity in T1-weighted imaging, and the capsule wall showed visible enhancement (Fig. 1E–1I). Endoscopic ultrasonography (EUS) indicated a unilocular cystic solid lesion in the pancreatic tail and the cyst was not found to communicate with the main pancreatic duct (Fig. 2A and 2B). Contrast-enhanced EUS using sulfur hexafluoride microbubbles showed that the solid component was enhanced in the arterial phase (Fig. 2C and 2D). Additionally, a EUS-based fine-needle aspiration (FNA) using a 22-G needle from the solid component was performed. The FNA sample consisted predominantly of leukocytes and proteinaceous debris, and no squamous epithelial cells or malignant cells were found.

**Figure 3:**
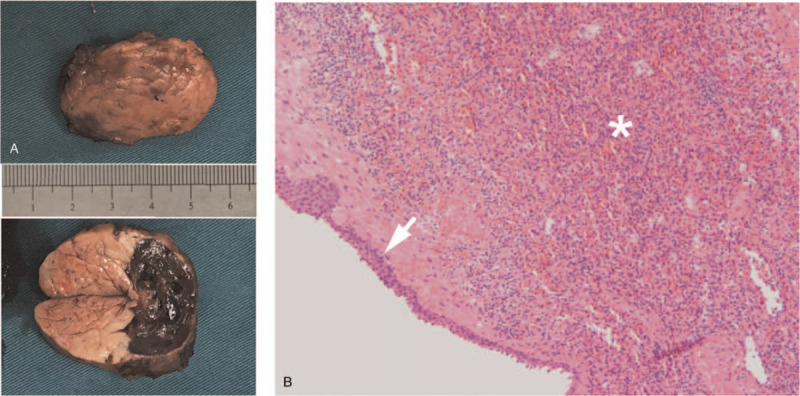
(A) Gross appearance of the cystic mass located in the pancreatic tail. The multilocular cyst measuring 2.6 cm at its greatest diameter is surrounded by a brown solid component. (B) Histological features of the pancreatic cyst. Microscopic analysis shows the squamous epithelial (arrow) cyst is enclosed by abundant splenic sinusoids, splenic cords, and lymphoid tissues (asterisk), suggesting an epidermoid cyst arising in an intrapancreatic accessory spleen (hematoxylin and eosin, ×50).

Based on the radiographic and pathological findings, ECIPAS was speculated. Considering the symptoms and the difficulty to completely exclude the malignant tumor due to the elevation of CA19-9, laparoscopic spleen-preserving distal pancreatectomy was performed. The surgical specimen revealed a well-defined cystic mass, measuring 2.5 cm at its greatest diameter, located in the tail of the pancreas (Fig. 3A). The cut surface showed that the mass was composed of a cyst and brown solid spleen-like tissue (Fig. 3A). The microscopic analysis demonstrated that the solid component was accessory splenic tissue, and the cyst wall was lined with a thin stratified squamous epithelium (Fig. 3B). The final pathological diagnosis was ECIPAS. The patient was discharged on day 5 postoperatively after an uneventful recovery. CA19-9 returned to normal after surgery. During a 2-years follow-up, there was no evidence of tumor recurrence.

## 3 Discussion

ECIPAS is an extremely rare entity. ECIPAS was thought to be benign until recently malignant transformation was found during a 6-years follow-up.^[10]^ Li et al^[11]^ reviewed 56 cases of ECIPAS since the first case was diagnosed by Davidson et al in 1980.^[12]^ Most of the cases were incidentally detected, while the others had symptoms of abdominal pain, discomfort, nausea, vomiting, back pain, fever, or weight loss. In all cases, cystic lesions were found in the pancreatic tail. The cysts could be either unilocular or multilocular, lined by a keratinized or nonkeratinized stratified squamous epithelium or a cuboidal epithelium. The average cyst size was 4.3 cm (range 1.3–15 cm). Although most cases of ECIPAS were benign, it was necessary to differentiate it from other potentially malignant pancreatic tail cystic neoplasms, including MCN, SPT, pseudocyst, IPMN, and cystic pancreatic neuroendocrine tumor (p-NET).

Elevation of serum CA19-9 level was common in ECIPAS patients. Hu et al reported that nearly 40% of ECIPAS showed high levels of CA19-9,^[5,13]^ hence increasing the difficulty to distinguish ECIPAS from malignant tumors preoperatively. It has been reported that the squamous epithelial lining of ECIPAS expressed CA19-9 in immunohistochemical analysis, and serum CA19-9 levels markedly decreased to normal levels postoperatively in patients.^[14]^ These findings suggest that serum CA19-9 is secreted by the epithelial lining cells of ECIPAS. In the current case, although ECIPAS was speculated, the symptoms and high CA19-9 level encouraged us to perform surgery.

Preoperative diagnosis of ECIPAS is difficult. MCN, cystadenocarcinoma, pseudocyst, cystic p-NET, or potential malignant tumor is suspected in most cases.^[15–18]^. Including the present case, only 6 (10.7%) among the 56 reported cases were correctly diagnosed preoperatively.^[15,19–22]^ Most cases of ECIPAS were diagnosed after surgery based on pathological findings.^[23]^ Advances in imaging techniques facilitated the diagnosis of ECIPAS as compared with previously; however, few studies have reported the imaging characteristics of ECIPAS. ECIPAS is a well-defined, unilocular, or multilocular cystic mass located in the tail of the pancreas on multimodality imaging. The well-defined boundary is a differentiating morphological feature suggestive of a benign tumor. The cystic wall of ECIPAS showed contrast enhancement similar to that of the spleen during multiphasic CT or MRI. Therefore, the accessory spleen surrounding the cyst was a key component for correct diagnosis. However, only a few cases of ECIPAS had a sufficient solid component that allowed splenic tissues to be detected through radiological imaging. Fortunately, there was a large amount of solid tissue present in our case; therefore, a correct preoperative diagnosis of ECIPAS was achieved. Including the present case, 4 of 56 cases were diagnosed as ECIPAS preoperatively based on the similar density on enhanced CT and intensity on MRI between the solid component and the spleen.^[15,20,21]^ The cystic component of ECIPAS usually appears to be hypodense on nonenhanced CT, hypointense on T1-weighted imaging, and hyperintense on T2-weighted imaging. However, it sometimes appears hyperdense on nonenhanced CT and hyperintense on T1-weighted imaging, in the presence of hemorrhage or keratinized materials within the cyst.^[5,24,25]^ In the present case, the cystic component was hyperdense on nonenhanced CT and hyperintense on T1-weighted imaging, and remained unenhanced during multiphasic scans. Thus, the cystic content was considered to be mucinous or bloody liquid that was confirmed by the resected specimen following surgery.

EUS-FNA is a commonly used technique to evaluate pancreatic masses, and it has been investigated for diagnosis of ECIPAS. In Tatsas et al report, 3 of 6 cases of intrapancreatic accessory spleen were diagnosed successfully; however, 1 case of ECIPAS failed to be diagnosed by EUS-FNA.^[26]^ In the only case of ECIPAS diagnosed postoperatively, the FNA sample revealed only predominant macrophages and proteinaceous debris; therefore, no pathological evidence of ECIPAS was acquired prior to surgery.^[26]^ Among the remaining 5 cases of ECIPAS reported in the English-language literature, 1 was correctly diagnosed by EUS-FNA.^[17,21,27–29]^ Matsumoto et al reported a case of ECIPAS that was accurately diagnosed by EUS-FNA.^[21]^ Their histological findings showed that sinusoids and abundant polymorphous lymphocytes were consistent with an intrapancreatic accessory spleen.^[21]^ Therefore, acquiring pathological evidence of ECIPAS using EUS-FNA seems to be difficult. This is mainly because the amount of splenic tissue surrounding the epidermoid cyst was too small in most cases to be successfully aspirated by FNA. In the present case, although leukocytes were found, we failed to reveal splenic sinusoids and endothelial cells within the acquired specimen.

Treatment of ECIPAS consists of surgical resection and follow-up. As most cases of ECIPAS are reported not to have malignant potential, unnecessary surgery can be avoided with a correct preoperative diagnosis. However, in cases of ECIPAS with symptoms, or in those in which it is difficult to completely exclude malignancy, laparoscopic spleen-preserving distal pancreatectomy is suggested since this procedure has been commonly used to treat benign or low-grade malignant tumors of the pancreatic tail. Fujii et al^[30]^ reported that laparoscopic distal pancreatectomy could be a useful, minimally invasive surgical approach for treating ECIPAS with additionally decreased postoperative pain, length of stay, and associated mortality and morbidity. In the present case, the symptoms and serum CA19-9 level encouraged us to perform surgery on this patient. The patient recovered uneventfully and had no evidence of tumor recurrence during a 2-years follow-up, suggesting laparoscopic spleen-preserving distal pancreatectomy is effective and safe for ECIPAS.

In conclusion, it is difficult to make an exact diagnosis of pancreatic tail cystic lesions preoperatively. ECIPAS is an exceedingly rare entity, and all cases reported so far have been found in the pancreatic tail. Therefore, although rare, ECIPAS should be considered in the differential diagnosis of pancreatic tail cystic lesions. Imaging and endoscopic evaluation may be helpful in the diagnosis, but preoperative diagnosis is still challenging. A similar enhancement between the solid component of the cystic lesions and the splenic parenchyma is critical for correct diagnosis. Treatment of ECIPAS by laparoscopic distal pancreatectomy is an effective procedure with the advantages of minimal invasiveness.

## Acknowledgments

The patient signed a written informed consent form for the purpose of publication of the results of this case study.

## Author contributions

XZ reviewed the literature, acquired the data, and contributed to manuscript drafting; BZ analyzed and interpreted the pathological and immunohistochemical findings, and contributed to manuscript drafting; QJS and MJ analyzed and interpreted the imaging findings and contributed to manuscript drafting; SY acquired the data and was responsible for the revision of the manuscript for important intellectual content; all authors issued final approval for the version to be submitted.

**Conceptualization:** Sheng Yan.

**Data curation:** Xiang Zheng.

**Writing – original draft:** Xiang Zheng, Ming Jin.

**Writing – review & editing:** Bo Zhou, Qing jing Sun.
